# The patient representation struggle during the COVID‐19 pandemic: Missed opportunities for resilient healthcare systems

**DOI:** 10.1111/hex.13877

**Published:** 2023-10-09

**Authors:** Hester van de Bovenkamp, Bert de Graaff, Karin Kalthoff, Roland Bal

**Affiliations:** ^1^ Erasmus School of Health Policy & Management Erasmus University Rotterdam The Netherlands; ^2^ Zorgbelang Inclusief Arnhem The Netherlands

**Keywords:** client councils, pandemic decision‐making, patient organizations, patient representation, qualitative research, resilient healthcare systems

## Abstract

**Background:**

The role of patient participation and representation during crises, such as the COVID‐19 pandemic, has been under‐researched. Existing studies paint a pessimistic picture of patient representation during the pandemic. However, there are indications that patient representatives have adapted to the new situation and can contribute to the resilience of healthcare systems. This paper aims to further explore the potential contribution of patient representatives for healthcare system resilience during the COVID‐19 pandemic.

**Methods:**

The study used a qualitative approach. We conducted a thematic analysis on the following data: interviews with client council members (*n* = 32) and representatives from patient organizations (*n* = 6) and focus groups (*n* = 2) to investigate patient representation on both the national policy level and organizational level in the Netherlands.

**Results:**

We identified the crisis discourse, the dependent position, the diversity of patient perspectives and the layered decision‐making structure as themes that help to understand what made patient representation in pandemic times a struggle for national and local patient representatives. The analysis of the subjects these representatives put forward during decision‐making shows that their input can play an important role in broadening discussions, challenging decisions, and suggesting alternatives during a crisis. We identified several strategies (e.g., collaborating with other actors, proactively putting subjects on the policy agenda, finding new ways of contacting their ‘constituency’) used by the patient representatives studied to exert influence despite the difficulties encountered.

**Conclusions:**

The struggle for patient representation during pandemic decision‐making is a missed opportunity for resilient healthcare systems as these representatives can play a role in opening up discussions and putting different perspectives to the fore. Moreover, the adaptive strategies used by representatives to influence decision‐making offer lessons for future representation activities. However, adaptations to the crisis decision‐making structure are also needed to enable patient representatives to play their role.

**Patient Contribution:**

We conducted interviews with patient representatives and discussed our preliminary findings with patient representatives during the focus groups. Zorgbelang, a patient organization supporting client councils and enabling and organizing patient participation for organizations and municipalities, was partner in this research and contributed to the interview guide, conducting interviews and focus groups. Additionally, the analysis made by the first author was discussed and refined multiple times with the partners of Zorgbelang and one of them co‐authored this paper.

## 1 INTRODUCTION

For some decades already, patient participation and representation in healthcare decision‐making has been on the policy agenda.^1^ In many countries, numerous initiatives have been taken to put participation and representation into practice in all kinds of decision‐making processes, including government policy‐making, research agenda‐setting and organizational policy‐making and quality improvement. Patients and their representatives (which can be patients themselves or representatives of patient organizations) are asked to contribute patient perspectives and represent their interests in these decision‐making processes. The aim of this participation and representation is to improve the quality of decision‐making as well as making it more democratic.^2^, ^3^ Despite this longtime recognition of its importance, the search for how to do participation and representation well and how to make it fitting to specific contexts continues.^2^, ^4^, ^5^

Participation and representation of patients in the context of crisis has been little researched so far. Several papers on participation and representation during the COVID‐19 pandemic that do exist paint a gloomy picture.^6^, ^7^, ^8^, ^9^, ^10^, ^11^, ^12^ Conclusions include that during crises it becomes clear that ‘we do not practice what we preach’ as far as patient participation is concerned^6^ and that ‘nothing about us without us was left hanging in the breeze’.^7^ The literature also includes some positive signals though. Shih et al.,^10^ for example, show that because the role of patient representatives became limited during the pandemic, which indeed shows ‘its brittle nature’, patient representatives searched for alternative strategies to influence decision‐making such as building new networks and lobbying. The pandemic therefore enabled representatives to reimagine their role, the authors conclude. They did so in a way that enabled them to translate the voices of the community to the healthcare system.

The adaptability shown by patient and client representatives is a key feature of resilient healthcare systems.^10^ Resilience and adaptability are seen as important aspects for responding well to crises such as pandemics.^10^, ^13^, ^14^, ^15^ Resilience is often conceptualized as an ability (of individuals, organizations or systems), or (measurable) outcome to adapt and ‘to bounce back’.^16^ We, however, argue that resilience in healthcare is not a stable state or something that can be achieved, but rather consists of continuous and concrete, mundane practices to cope with unforeseen circumstances. To conceptualize resilience in healthcare as context‐dependent and practice‐based, is rather new, but follows from analyses of the pandemic responses.^17^ Research and evaluations of how the pandemic was governed often point to the need for increasing community participation.^17^ Reflexivity and being open to learn from different perspectives and types of knowledge are considered crucial aspects for fostering the adaptive practices in healthcare needed to foster resilience.^13^, ^18^ In this paper we dive further into the potential contribution of patient representatives for healthcare system resilience.

The Netherlands is an interesting case to study as patient representation has been institutionalized on both organizational and national policy levels for decades now.^1^, ^19^ Since the 1980s, the Dutch government has actively encouraged patient representation in decision‐making.^20^ Interestingly, participation has become highly formalized. Patient organizations are asked to participate in formal decision‐making processes as patient representatives. The Dutch government has enabled patient organizations to play this role by awarding subsidies and by opening decision‐making processes, such as with respect to medical guideline development, research agenda‐setting, and government policy‐making.^20^, ^21^ In healthcare organizations, client councils play an institutionalized role representing patients. These councils have become mandatory under the Co‐Determination of Health Care Institutions Act (WMCZ).

The question if and how this representation holds up during times of crisis, and if it indeed can contribute to practices of healthcare system resilience is therefore interesting to explore. We do so by answering the following research question: *How did patient representation take place during the COVID‐19 pandemic in the Netherlands and what does this mean for its contribution to the resilience of the healthcare system?*

In this paper we show that despite its institutionalization, also in the Netherlands patient representation was under pressure in pandemic times. We identify several factors that contributed to this situation. We also show the potential of patient representation for practicing resilient healthcare systems. In the discussion we elaborate on lessons for the future of patient representation during crises and identity points for further research.

## 2 METHODS

Our study into patient representation during the COVID‐19 pandemic in the Netherlands was part of a larger multisited ethnographic study of Dutch healthcare governance during the pandemic.^22^ The Dutch healthcare system is governed through a layered decision‐making structure. This was especially so during the pandemic.^13^, ^23^ During this time an attempt was made to balance a process of centralizing crisis decision‐making with consensus‐based decision‐making with relevant and prominent stakeholders, such as medical professionals and virologists.^24^ This balancing act coincided with the existing system of regulated competition between healthcare providers, and in particular the regional level was instrumentalized as the main site to implement mitigating measures.

Our study focused on these different levels of decision‐making and the different actors involved. The study started in March 2020 in a specific region in the Netherlands and, from September 2020, expanded to include two more regions and national level stakeholders as well. We elaborate on this broader study elsewhere.^25^ One of the focus points of our research was the question if and how patients were represented in decision‐making. To learn more about patient representation during the pandemic we conducted a more focused qualitative mixed‐method study in which we wanted to learn more about representation on different levels of crisis decision‐making. Zorgbelang, an organization supporting client councils, enabling and organizing patient participation for organizations and municipalities, was partner in this research and contributed to the interview guide, conducting interviews and focus groups.

We conducted our qualitative study on patient representation during the COVID‐19 pandemic on both the national policy level (focusing on national patient organizations aiming to influence national COVID‐19 policy‐making) as well as the organizational level (focusing on client councils aiming to influence healthcare provider policies in response to the pandemic) in the Netherlands. The national level was an important level of decision‐making as COVID‐19 decision‐making was for an important part happening top‐down. Here, we expected patient organizations to play an important role in representing patients’ interests as they are involved during government decision‐making during ‘normal’ times. In the Netherlands the regional level was also an important level of crisis‐decision making.^24^ However, here patient representatives proved largely absent (more on this in the results section). To get an impression of local representation we also included the organizational level, as healthcare provider organizations also had to make important decisions on how to respond to the pandemic.

### 2.1 Interviews

First, we conducted interviews with representatives from the national and organizational (healthcare provider) levels of decision‐making. We conducted 23 interviews with client councils, sometimes multiple members of these councils were present (a total of 32 members participated during these interviews). We selected members from different healthcare sectors, including older person care, care for people with mental health problems and mental disabilities and hospital care. This selection was made to get an overview of patient representation on the organizational level in different sectors with the possibility of identifying important differences between them. We selected client councils from the three regions that were the focus of our broader study. These regions were purposively selected based on the severity of the caseload and the composition of the acute care sector. In addition, we interviewed 6 patient representatives from patient representation organizations active on the national level. We again selected respondents focusing on representation in different sectors; curative care, older person care, mental healthcare and care for people with disabilities.

Interviews were semi‐structured and included questions about the topics respondents felt to be important during COVID decision‐making, the extent to which they felt they could influence decision‐making and what determined this, what strategies they used to influence national or organizational decision‐making and how they contacted their ‘constituency’ to determine their position and to account for their representation work. Interviews were recorded and transcribed verbatim. The interview guide is added as a Supporting information.

### 2.2 Focus groups

Second, we conducted two focus groups. One with client council members (*n* = 6) and one with respondents from national patient organizations (*n* = 6). We contacted the organizations and councils who participated in the interview phase and said that they would be willing to contribute to the next phase of the research. In addition, we contacted two additional national organizations suggested by other respondents to complement their input. We started these focus groups with a short presentation on the results of the analysis of the interview data to validate our results. We discussed if these results were recognizable and if respondents, based on their experiences, missed important themes. This led to broadening up certain themes, such as the subjects and groups patient representatives focused on during the pandemic and their attempts to meet their constituency. We then moved on to discuss respondents’ experiences and ideas about the role of patient representation during the pandemic and what was needed to strengthen this role. The focus groups were also recorded and transcribed verbatim.

### 2.3 Analysis

We conducted a thematic analysis of the interview and focus group data.^26^ This analysis was a combination of an inductive and deductive approach as these themes were partly grounded in the findings of our broader study.^25^ The analysis came to focus on the themes of the language of ‘crisis’ and re‐framing, representatives’ dependent position, the diversity of their perspectives, the layered decision‐making structure, and strategies representatives used to influence decision‐making.

We followed different analytical steps, largely identified by Braun and Clarke,^26^ but with some extra steps built in as a result of the additional data gathered during the focus groups and the contextualization of the analysis based on the broader project. First, the first author became familiar with the data by reading the interview transcripts. Next, initial codes were generated staying close to the wording of respondents. In the next step the first and third author pulled together these codes into broader themes. These themes were reviewed and refined multiple times during discussions with the partners of Zorgbelang who conducted the interviews with client councils. Discussions centred on identifying the most important themes and exploring the specificities of these themes (e.g., the different role perceptions of client councils). The themes from the analysis of the interview data were reviewed further by presenting and discussing them at the start of the focus groups to validate and extent our analysis. Our analysis was then further refined by a thematic analysis of the focus group data. This analysis was conducted by the first author by thematically coding the transcripts of the focus groups, using the themes defined in the previous steps. Subsequently, a thematic summary was made of this analysis, which was reviewed and discussed with the other authors. Themes from the initial analysis of the interview data were used and further refined during this process (e.g., the theme on differences in patient perspectives and ‘forgotten’ groups). Additionally, the theme of possible improvement in representation in future crisis decision‐making was extended (e.g., on the need to limit fragmentation of decision‐making). We then wrote up our analysis. During the writing process, the analysis presented in the paper has been discussed and refined based on multiple discussions amongst the authors involved in this paper.

### 2.4 Ethics

Our project was approved by the Research Ethics Review Committee of Erasmus School of Health Policy and Management (21‐009). We obtained prior and explicit consent from the participants of the interviews and focus groups. The quotes used in this paper have been anonymized.

## 3 RESULTS

In this section we first discuss the struggle of patient representation focusing on different factors that contributed to this struggle according to our respondents. Next, we describe the potential of representation work for broadening discussions, challenging decisions, and suggesting alternatives during crisis decision‐making. Third and finally, we analyse the strategies used by patient representatives to influence decision‐making despite the difficulties encountered.

### 3.1 The patient representation struggle

In our study we identify four main themes that help explain what made patient representation a struggle during the pandemic. We discuss them in turn but would like to note that these themes interact to a large extent.

#### 3.1.1 ‘It is a crisis!’

First, the dominant crisis discourse made it difficult for patient representatives to make their voices heard. Calling on ‘the crisis situation’ implied, especially in the first phase of the pandemic, that there was no or little time to consult representatives. The crisis discourse meant that the usual decision‐making procedures were put aside in favour of often more top‐down and ad‐hoc crisis‐decision‐making. This resulted in a national patient organization with a long‐standing position in healthcare decision‐making having to first fight themselves in.

I think at the very beginning, it very much felt like we were being left out. So, suddenly, we were an observant of what was happening. And sometimes we were informed about what was going on, and then we really, I think that was during the first weeks, we had to raise awareness like: hello, normally we are a partner, and where are we now? And then we were invited to all the meetings. And then we were in our normal role, but that is very generally stated. Because what you see is that during that crisis it is just about one thing only: are there enough beds available? Do I have to move patients? And the rest kind of follows from that. (interview respondent national patient organization)

After this initial struggle this organization was included in consultation procedures initiated by the Ministry of Health, but, as we will show below, the struggle to influence decision‐making was not over.

The crisis discourse also played a role on the level of healthcare organizations, limiting the role of client councils. Importantly here, this discourse was not only used by other actors. Several client councils also used it themselves and concluded that in times of crisis, others, including them, should not interfere in decision‐making.

… in case of crisis management, then the other should not interfere, that includes us as a client council. (client council member hospital)

Moreover, the dominant way of framing ‘the crisis’ as a crisis of infection rates and acute care also made it difficult for patient representatives to influence decision‐making. Because of this focus it proved near impossible to put issues in long‐term care or mental health firmly on the national agenda. ‘The real important decisions were made based on other sectors’ as one of our respondents active on the national level explained during a focus group. But even national representatives focusing on curative care had a hard time putting other subjects, such as the delay of regular ‘non‐COVID’ care, on the agenda.

#### 3.1.2 A dependent position

The crisis discourse as a mechanism for exclusion played a prominent role in the first phase of the pandemic. This became less so when time passed, although it remained a struggle for the patient representatives we studied to influence decision‐making, in part because of the dominant frame of the crisis described above. Also, dependence on other actors remained high.

In the case of national representatives, we saw that when they were included in the discussion, the dependent position meant that influence was still very limited indeed. This led to the danger of instrumental use of their participation. For instance, a national representative during our focus group stated that their participation could be used to legitimize decision‐making when the COVID‐19 measures were discussed in other fora:

because the moment [a member of Parliament] asks a question in the debate, the minister says yes, but we have consultations with them [patient representatives] once every two weeks. (focus group national patient organizations)

In the case of client councils, the possibility to influence decision‐making was largely determined by Boards of Directors of the healthcare provider and department and location managers. This could severely limit the space to play a representative role because it could be that no or limited space was offered to discuss the measures taken. This limited role was exacerbated during the beginning of the pandemic as many council members were denied access to the healthcare organization as part of the restrictive measures. There were also council members who were afraid of becoming infected themselves and therefore did not want to meet. This restricted access meant that client councils could not have meetings, nor could they easily meet the people they represent. Especially in older person care and care for people with mental disabilities, members had difficulty in switching to digital meetings.

#### 3.1.3 Diversity of patient perspectives

Governing the pandemic meant having to deal with conflicting interests. These include conflicting interests between different care sectors and groups of patients. This relates to the fact that there is no such thing as ‘the patient perspective’. Representation of patients is, by definition, diverse in nature. It matters if you are an older person living in a nursing home, a young person with mental disabilities living in a healthcare facility, an acute COVID‐19‐patient or a patient made to wait for a procedure. The consequence was that when representatives were included in the decision‐making process, conflicting interests were put forward. Respondents from the national level noted that some perspectives were more dominant than others at decision‐making tables.

We were also involved in a consultation in the beginning (…) which was for the elderly and people with a disability or chronic illness, and we noticed that the elderly in particular were very dominant in the discussion, which meant that people with a disability due to a chronic illness, especially people who live at home, who receive care and support there, were not really considered at all. Those were not in the picture at all. (focus group national patient organizations)

The dominance of certain groups and differences in the amount of attention paid to certain groups and sectors during the pandemic magnified pre‐existing differences, according to our respondents.

National organizations also noted differences in opinion within their constituency which made it important to explain their reasoning well. They also pointed to the importance of local representation in this regard as this would enable representational work to be adjusted to local contexts. Diversity of patient perspectives was indeed also present locally. For example, while some client councils focused on trying to open‐up organizations as much as possible, calling on the importance of quality of life, there were also councils proposing stringent measures to limit infection rates as much as possible. As contact with the constituency also was limited during the pandemic this was, it seems, in large part dependent on the individuals active in the councils.

#### 3.1.4 A layered decision‐making structure

The layered nature of patient representation on the national and local level can be considered important to include voices of patient representatives in a general sense and do justice to local contexts. However, we also saw that the layered nature of COVID‐19 decision‐making was a factor contributing to the struggle of including patients’ voices.^13^, ^23^, ^24^ This layeredness meant that patient representatives were not or only to a limited extent present in decision‐making fora that played an important role in the governance of the pandemic. At the national level, patient representatives were not present in important advisory councils, the Outbreak Management Team being an important one. This team, consisting of medical experts and infectious disease experts, played a vital role in the governance of the pandemic, advising the government on what kind of restrictive measures to take. Patient representatives were also largely absent at the regional level:

We caught much less of what was going on there [regional decision‐making]. We sometimes raised a finger and said there should also be patients [there]. Well, you could forget that. (respondent national patient organization)

Respondents analysed being absent at different tables that played an important role in the governance of the pandemic as ‘playing football on the wrong field’ or being at the ‘side table’. The fact that representation at the regional level was very limited also caused problems at other levels of representation. Client councils in hospitals for instance noted that they, as did the hospital itself, were presented with decisions already taken at the regional or national levels.

One of the problems resulting from this layered and fragmented decision‐making structure was limited reflexivity on the different and perhaps missing perspectives relevant for crisis decision‐making. In the words of one of our respondents:

So what you noticed in the discussion and what you might be able to avoid if you do sit down together is that quality of life is a trade‐off with infection prevention. Nobody wants to get heartily ill from corona of course. And the very conversation about that can be of ‘gee, what do we find an appropriate direction that weighs both perspectives?’ Then these different football fields don't help, then it's either about this or about that. But it has to be about both to eventually decide how to deal with this. (focus group national patient organizations)

To conclude, we identified different themes—a dominant crisis discourse, the dependent position, the diversity of patient perspectives and the layered decision‐making structure—that help to understand what made patient representation in pandemic times a struggle for national and local patient representatives. Especially the dominant crisis discourse and hence the role of language proved pivotal in this struggle. The question is, as some client councils themselves asked, if this should be considered problematic. In the next section we show that this can indeed be considered problematic by showing instances where patient representatives were trying to reframe the crisis, thereby showing their potential for contributing to broadening perspectives in decision‐making.

### 3.2 Reframing the crisis: Questioning dominant policy discourse

Our study shows that patient representatives can play an important role in broadening discussions, challenging dominant discourse, and suggesting alternatives during a crisis. From this perspective, the diversity of perspectives put forward by different representatives can actually be seen as an advantage, as it helps to open up discussions and challenge the dominant framing of the crisis.

For example, representatives on the national and local level called for attention to quality of life in addition to and sometimes at the expense of a mere focus on infection control. Clients’ freedom and the psychological damage of the measures taken, such as banning family visits, are key concerns in this regard:

Actually, that's kind of weird that you call family ‘visitors’. They are people who belong together. And because of that [calling them visitors] you reduce them to someone who comes for a cup of tea once in a while […]. (respondent national patient organization)

There were also representatives who drew attention to the adverse effects of the measures on the (mental) health of citizens. A national patient organization focused on curative care for example continued to call for attention for care that was delayed because of the focus on acute COVID‐care.

Because we also felt like this is really irresponsible how everything else is just turned off like that. (respondent national patient organization).

And the umbrella organization for people with mental health problems called for attention to specific groups to which little attention was paid during decision‐making, and for whom exceptions to certain rules were needed:

We have quite some people in our constituency who completely loose it at the moment they need to wear a facemask. Then at some point you need to find an exception for that. (respondent national patient organization)

Reasoning from the perspective that if certain measures cannot be adapted, it was important to ‘regulate things around them’, the focus of several representatives was on reducing the negative consequences of the measures aimed at reducing infections. Examples include pushing the boundaries of what could still be done concerning activities in long‐term care or building tents outside the hospital for waiting family and friends of patients who were not allowed inside. The way measures were communicated was also an important focus point for some client councils, critiquing the distant language used in letters to family members and making sure they became more compassionate:

I think that was also with one of the letters. Like, you don't send this out like this (interview client council member elder care).

The above shows that dominant assumptions underlying measures taken to limit the spread of the virus were questioned and, sometimes, successfully challenged by emphasizing and framing the crisis in terms of quality of life. Moreover, alternatives were put forward or attention was asked for the effects of measures for groups of patients who were considered too little in the decision‐making according to our respondents.

### 3.3 Strategies used to influence decision‐making

We saw that it was not easy for patient organizations and client councils to influence COVID‐19 decision‐making, which can be seen as detrimental from the perspective of resilient healthcare systems as their input could be used to adapt to changing circumstances taking on board multiple perspectives. However, in line with Shih et al.^10^ we identified several strategies used by the patient representatives studied to exert influence and reframe discussions despite this and to work around the complicating factors we identified.

Collaboration with other parties in the healthcare field was one such strategy. For example, on the national level patient representatives cooperated with other actors, such as branch associations, to arrange things themselves outside the formal national consultation tables. The development of guidelines for visiting arrangements was one example. Cooperation with regulators by passing on signals patient organizations received from their members or by jointly calling attention to specific subjects such as postponed care, are others. At the national level, patient organizations also pulled together at times in their advocacy activities. This cooperation was described by one representative as a ‘bright spot’ of the crisis.

Another strategy to exert influence was to proactively put certain subjects on the policy agenda, both on the national and organizational level. For example, patient organizations drew attention to certain issues, such as postponed care in the media, or in letters to Parliament. There were also some proactive client councils that gave Boards unsolicited advice or questioned them critically. Earlier we identified client councils who moved to the backseat of decision‐making because they did not want to be in the way in times of crisis. However, there were also client councils reasoning the opposite way and became more pro‐active. Stating ‘we basically deal with everything that is very close to the residents’ (Interview client council member long‐term care), they reasoned that client councils have an important role to play precisely at the time of the pandemic because the measures being taken to control the virus had such a large impact on people's lives. This led to a critical stance to the measures being taken to control the spread of the virus:

Well, that did lead to discussions. Also because sometimes you are diametrically opposed to each other, because what is more important? Is health and the outbreak of corona the main issue, or is it the mental state of your client?(respondent client council)

Interestingly, we only found client councils in long term care reasoning from this perspective; there were no client councils from hospitals that took such a pro‐active stance.

There were also councils that developed short lines of communication with directors through informal contact and tried to adjust policies in this way. The switch to digital meetings and digital contact with managers provided opportunities for this. So when (and if) client councils adjusted to this way of working this was also put to their advantage. Several respondents also noted the advantages of this for strengthening their position in the future.

In addition, we saw that several representatives continued to focus on topics that were important to them besides issues related to the pandemic. One example is councils that continued their ‘regular’ advisory work. Other representatives championed the opportunities COVID‐19 presented for certain changes in care in ‘normal’ times, such as the accelerated introduction of digital care.

Importantly, there were also representatives that tried to find alternative ways to contact their ‘constituency’. As we already noted, this contact was more difficult during the pandemic as these measures meant that client council members could not enter the healthcare organizations at different times during the pandemic. Some sought ways to combat this, for example by standing at the entrance of the organization to talk to family members or contact persons within the organization. National organizations arranged contact points and conducted research amongst patients to learn more about their experiences which they used to determine their representation work.

These examples show that also in the Dutch case there are patient representatives who can adapt themselves to changing circumstances. Respondents noted that this adaptability was partly dependent on the people involved, which does make it fragile:

That means there is a kind of institutional vulnerability that has to be compensated by personal strength (focus group on national representation).

## 4 DISCUSSION

Our findings show, in line with earlier research, that the representation of patients in collective decision‐making during the pandemic was under pressure.^6^, ^8^, ^10^, ^11^ This was the case also in the Netherlands even though here representation has been institutionalized for decades. We identified different themes—the dominating crisis discourse with a specific framing of the crisis in terms of infection control and acute care, the dependent position, the diversity of patient perspectives and the layered decision‐making structure—that contribute to this.

As reflexivity and being open to learn from different perspectives and types of knowledge can be considered crucial aspects for the adaptive practices needed to foster resilience, we can also conclude that patient representatives can potentially contribute to resilient healthcare systems. We identified that by attempting to open‐up dominant discussions and calling for attention for negative consequences of certain policies or forgotten groups patient representatives can contribute to resilient practices in healthcare. Moreover, we saw that representatives were able to adapt and use different strategies to bring these issues to the fore showing their own resilience in times of crisis.

### 4.1 Lessons for and about patient representation during crisis

Following our analysis, and in dialogue with existing literature, we draw out several lessons for patient representation during crisis which we summarize in Table 1 and detail below.

**Table 1:** Key lessons about patient representation in crisis governance.

Patient and client organizations have a role to play in resilient crisis management practices, but much now depends on individuals
The input of patient representatives in crisis governance can help to discuss and challenge dominant frames of the crisis.
Diversity of patients’ perspectives can be regarded as an asset but requires work and reflection to be put to use.
Different patient representatives need to connect on and between different decision‐making layers and relate to each other and other actors in the crisis governance system.
Patient representation needs to adapt to ad hoc, emerging, crisis governance structures.

Recent literature increasingly recognizes the dynamic and mundane practical nature of resilience in healthcare and the roles that community groups play in resilient responses to crises^27^; patient representatives most certainly have a role to play here, for instance in fostering reflexivity and learning.^13^, ^18^ The ability of patient representatives to play this role can also be seen in the case of the recognition and care for Long‐COVID. By sharing stories on social media about long‐term complaints after an infection they succeeded in the recognition for this condition.^28^, ^29^

Moreover, crisis management literature sees crisis‐responses as a ‘self‐organizing response’ in a myriad of emerging and adaptive practices.^30^ We saw that on both the national and the organizational (provider) levels there were indeed representatives that were able to adapt to the new situation, build alternative strategies to influence decision‐making and to contact those they claimed to represent. However, especially on the organizational level this seemed to depend on the individuals involved and the informal relations in place. Their alternative strategies we identify (see Table 2) can serve as important lessons for the future of representation, perhaps also in ‘normal’ times.

**Table 2:** Key strategies for practicing patient representation in crisis governance.

Reframing the dominant issue at stake
Collaborating with other parties
Pro‐active agenda‐setting
Engaging in informal communication with decision‐makers
Finding alternative ways to contact constituencies

The ability to broaden discussions and bring different perspectives to the fore is important especially during times of crisis in which the tendency is doing the opposite; namely arguing for de‐politization and letting ‘the experts’ decide. This tendency results in specific frames of the crisis to become dominant.^25^ As these frames emphasize certain values at the cost of others they are in fact highly political in nature.^31^ In case of the pandemic the dominant frame of the crisis in the Netherlands focused on infection prevention and the consequences of the pandemic for acute care. The input of patient representatives in crisis decision‐making can help to discuss and challenge these frames. The diversity of patient perspectives can be regarded as an asset in this regard as insight into this diversity enables the weighing of different interests in decision‐making. The wish of policy‐makers for a ‘unified patient perspective’^20^ might be understandable in terms of efficiency, when it comes to a wish for more reflexive decision‐making it can be considered counterproductive. However, putting this diversity to good use is not a given as we find how certain patient groups dominated crisis governance and how differences in the amount of attention paid to certain groups and sectors during the pandemic magnified pre‐existing differences.

An important aspect of re‐thinking their future role is how different representatives on and between different decision‐making layers relate to each other and other actors in the governance system. To make these connections well the representation of patients should be discussed and organized also on the levels and within the fora they were now largely absent from, but which played a dominant role in the crisis‐decision‐making. Hence, adaptations to the emerging crisis decision‐making structure during a crisis and (in)formal networks and practices that are now being put in place to prepare or prevent crises from occurring is also needed to enable patient representatives to play their role.

### 4.2 Limitations and future research

Our empirical study was focused on the Netherlands, hence limiting the transferability of our findings. However, we would argue that considering the high level of institutionalization of patient representation in Dutch healthcare our study might be considered a ‘most‐likely’ case‐study for patient representation to play an important role in comparison to other healthcare systems. Moreover, we only focused on a selection of sectors in Dutch healthcare. Future research might emphasize observations of representative practices and the exploration of other parties that interact with patients and claim their representation. Finally, as patient representation is a layered and fragmented practice, future research could focus more on the patient representative system during and outside crises by including different levels of decision‐making and their formal and informal connections and on different actors making representative claims.^32^

## 5 CONCLUSION

We conclude that patient representation by patient organizations and client councils proved a struggle during the pandemic in the Netherlands. Different factors—the crisis discourse with the dominant frame of infection prevention, the dependent position, the diversity of patient perspectives and the layered decision‐making structure—contributed to this. We conclude that this situation is a missed opportunity for resilient healthcare systems as these representatives can play a role in opening up discussions and putting different perspectives to the fore, thereby enhancing the potential for adaptability to the crisis. At the same time, we conclude that representatives proved to be adaptive themselves in exploring new strategies to influence decision‐making, which offers important lessons for the future of patient representation.

## AUTHOR CONTRIBUTIONS

**Hester M. van de Bovenkamp**: Study design; data collection; data analysis and interpretation; drafting and completing manuscript. **Bert de Graaff**: Study design; assisted in data analysis and interpretation; assisted in drafting and completing the manuscript. **Karin Kalthoff**: Study design; data collection; assisted in analysis and interpretation; assisted in drafting and completing the manuscript. **Roland Bal**: Study design; assisted in data analysis and interpretation; assisted in drafting and completing the manuscript. All authors read and approved the final manuscript.

## CONFLICT OF INTEREST STATEMENT

The authors declare no conflict of interest.

## Supporting information

Supporting information.

## Data Availability

The data that support the findings of this study are available on request from the corresponding author. The data are not publicly available due to privacy or ethical restrictions.
