# Supplementary material for: The patient representation struggle during the COVID‐19 pandemic: Missed opportunities for resilient healthcare systems
Source: Health Expect. 2023 Oct 9;27(1):e13877. doi: 10.1111/hex.13877 (PMC10768858; doi:10.1111/hex.13877)
Supplement: Supplementary file 1 — Supporting information. [file HEX-27-e13877-s001.docx]

**Topic lists used during interviews**

- What were the most important themes for your client council/patient organization during different stages of COVID-decision-making?
- How did you decide on these themes (e.g. contact with constituency, own experiences, contact with professionals )?

- How are you as a client council/patient organization involved in organizational/regional or national decision-making during different stages of COVID-decision-making?
- In what way did you try to influence organizational/regional/national policies? How does this differ from decision-making during ‘normal’ times?
- In what ways were you able to contribute to decision-making and in what ways? On what topics and in which stage? What were the reasons which made contributing to decision-making difficult?
- How did your contributions impact decision-making?
- How did you keep in contact with the groups you represent? How was this different compared to ‘normal’ times?
- Were there other actors that represented the interests of patients and advocated for the topics that were important to your council/organization and did you have contact with them?
